# Pioglitazone alleviates oxygen and glucose deprivation-induced injury by up-regulation of miR-454 in H9c2 cells

**DOI:** 10.22038/IJBMS.2018.29223.7063

**Published:** 2018-10

**Authors:** Nianzi Sun, Lin Yang, Qian Zhang, Chengwei Zou

**Affiliations:** 1 Shandong University, Jinan 250100, Shangdong, China; 2 Department of Cardiac Surgery, Linyi People’s Hospital, Linyi 276000, Shandong, China; 3 Department of Equipment, Linyi People’s Hospital, Linyi 276000, Shandong, China; 4 Department of Cardiac Surgery, Provincial Hospital Affiliated to Shandong University, Jinan 250021, Shandong, China

**Keywords:** ERK/MAPK, MicroRNA-454, Oxygen glucose deprivation Pioglitazone, PI3K/AKT

## Abstract

**Objective(s):**

Pioglitazone, an anti-diabetic agent, has been widely used to treat type II diabetes. However, the effect of pioglitazone on myocardial ischemia reperfusion injury (MIRI) is still unclear. Herein, the objective of this study is to learn about the regulation and mechanism of pioglitazone effects on oxygen glucose deprivation (OGD)-induced myocardial cell injury.

**Materials and Methods:**

A cellular injury model of OGD-treated H9c2 cells *in vitro *was constructed to simulate ischemic/reperfusion (I/R) injury. Then, various concentrations of pioglitazone (0, 2.5, 5, 7.5 and 10 μM) were used for the treatment of H9c2 cells, and CCK-8, flow cytometry and western blot assays were performed to examine cell viability, apoptosis, and the protein levels of factors involved in cell cycle and apoptosis in OGD-treated cells. MiR-454 inhibitor was used to suppress miR-454 expression, and whether miR-454 was involved in regulating OGD-induced cell injury was studied. Two key signal pathways were examined to uncover the underlying mechanism.

**Results:**

OGD reduced cell proliferation and induced apoptosis in H9c2 cells (*P*<0.05, *P*<0.01 or *P*< 0.001). OGD-induced injury was significantly attenuated by pioglitazone at the concentration of 5 μM. Additionally, pioglitazone significantly up-regulated miR-454 expression in OGD-injured cells (*P*< 0.05 or *P*< 0.01). MiR-454 suppression declined the protective effect of pioglitazone on OGD-injured H9c2 cells (*P*<0.05 or *P*< 0.01). Besides, pioglitazone activated PI3K/AKT and ERK/MAPK pathways via up-regulating miR-454.

**Conclusion:**

Pioglitazone protected H9c2 cells against OGD-induced injury through up-regulating miR-454, indicating a novel therapeutic strategy for treatment of MIRI.

## Introduction

Ischemia/reperfusion (I/R) injury is a common pathological and physiological phenomenon in clinic, which is the resultant from multiple factors (1). With the development of coronary intervention and thrombolytic therapy, myocardial ischemia reperfusion injury (MIRI) is becoming more and more common (2). Accumulating evidences have demonstrated that MIRI could induce arrhythmia, enlarge infarct size, and lead to ventricular systolic dysfunction (3, 4). MIRI has negative effect on human health, and it remains a challenge to alleviate MIRI for researchers and clinicians (5). Currently, conservative drug therapy is still considered to be effective and safe method for the treatment of MIRI (6). Therefore, it is of great significance to find a novel drug for the treatment of MIRI. 

Pioglitazone is a highly selective and powerful peroxisome proliferator-activated receptor-γ (PPARγ) agonist, which has been widely used to improve insulin resistance and control blood sugar in the clinic (7). In the recent years, pioglitazone has been reported to be linked with various diseases, such as ischemic stroke, dementia and bladder cancer (8-10). Moreover, several studies have demonstrated that pioglitazone has anti-inflammatory, and antioxidant stress effects; as well, it improves myocardial energy metabolism and inhibits cardiomyocyte apoptosis, thereby protecting cardiomyocytes (11-13). A rat model experiment from Li *et al*. reported that pioglitazone could protect cardiomyocytes through decreasing apoptosis in these cells, which subsequently reduces mitochondrial ultrastructure injury and membrane potential loss in the I/R heart of rat (14). Another study found that pioglitazone could alleviate MIRI through up-regulation of extracellular signal-regulated kinases (ERK) and cyclooxygenase (COX)-2 (15). Despite these studies have demonstrated the role of pioglitazone in MIRI, further exploration regarding the regulatory mechanisms is still necessary.

Accumulating evidences have uncovered that miRNAs are involved in various biological processes of the heart (16). Mounting evidences confirmed that miRNAs expression in cardiomyocytes was significantly changed after IRI, suggesting the important roles of miRNAs in MIRI (17, 18). The abnormal expression of miR-454 is found in various cancers, and is closely associated with lung injury (19, 20). However, the effect of miR-454 on MIRI remains unclear. The objective of this study is to investigate the protective effects of pioglitazone on MIRI in H9c2 cells. An oxygen glucose deprivation (OGD)-induced H9c2 cells injury model *in vitro* was firstly constructed. Moreover, the effect of miR-454 was examined on OGD-induced H9c2 cells injury. Mechanistically, the relevant signal pathways of phosphoinositide 3-kinase (PI3K)/protein kinase B (AKT) and ERK/ mitogen-activated protein kinase (MAPK) were investigated. These results might provide a novel therapeutic strategy for MIRI.

## Materials and Methods

***Cell culture and OGD-induced cell injury model construction***

The H9c2 cell line purchased from American Type Culture Collection (ATCC, Rockville, MD, USA) was used in the present study. For cell culture, the cell culture bottle of H9c2 cell line was opened under the sterile condition. 

The medium in the bottle was sucked out, and the cell surface was cleaned 2-3 times with phosphate-buffered saline (PBS). Then, the supernatant was discarded, and the cells were digested with 0.25% trypsin-EDTA (Gibco-BRL, Gaithersburg, MD, USA). When the cells were turned round, the complete medium was added to stop the reaction. The cells were then transferred to a new culture bottle, and the commonly-used Dulbecco’s modiﬁed Eagle medium (DMEM, LifeTechnologies, Carlsbad, CA, USA) containing 10% (v/v) fetal bovine serum (FBS, Gibco-BRL), 1% (v/v) Penicillin-Streptomycin double resistant (Gibco) and 1% (v/v) GlutaMAX (Life Technologies) was added to further culture cells under the routine condition. The culture medium was changed every 2-3 days. 

For OGD treatment, H9c2 cells were switched from high-glucose DMEM to free-glucose DMEM for one day treatment. Then, these cells were placed in an anaerobic condition with 5% (v/v) CO_2_ and 95% (v/v) N_2_ for 0, 2, 4, 6 and 8 hr; culture temperature was controlled at 37 ± 0.5°C. Subsequently, these cells were recovered to the conventional culture. H9c2 cells were cultured under normal conditions and served as a blank control group. pioglitazone purchased from Sigma-Aldrich (St Louis, MO, USA) (21) was configured at different concentrations of solution (0, 2.5, 5, 7.5 and 10 μM). H9c2 cells were pretreated with pioglitazone for 12 hr before accepting OGD stimulation. Furthermore, 5 μM pioglitazone was selected as the optimum concentration for the following research.

***Cell counting kit-8 (CCK-8) assay***

The cell proliferation/toxicity detection kit, CCK-8 (Dojindo Molecular Technologies, Gaithersburg, MD) was used to determine the cell viability of H9c2. Briefly, H9c2 cells were collected, and cell suspension concentration was adjusted to 5 × 10^3^ cells/well. Cells were cultured in 96-well plate at 37°C for 24 hr in an incubator containing 5% (v/v) CO_2_, and then different concentrations of pioglitazone (0, 2.5, 5, 7.5 and 10 μM) were added into 96-well plate for stimulating H9c2 cells for 12 hr. After treatment, 10 μl of CCK-8 solution was added to each plate well and continued to culture for 1 hr at 37°C in CO_2 _incubator. The 450 nm wavelength was chosen to measure the absorbance value by a Microplate Reader (Bio-Rad, Hercules, CA, USA).

***Apoptosis assay***

Annexin V-FITC/PI apoptosis detection kit (Beijing Biosea Biotechnology, Beijing, China) was used to examine the percentage of apoptotic cells of H9c2 cells. After treatment with 5 μM Pioglitazone, treated cells were collected and washed twice with pre-cold PBS. Subsequently, cells were re-suspended in 1 × binging buffer, and cell suspension concentration was adjusted to 1 × 10^6^ cells/well. Then, 100 μl cell suspension was added to the bottom of flow tube, and 10 μl Annexin-V and 5 μl propidium iodide (PI) were added to stain these cells for 15 min under the shading condition at room temperature. After this, cells were re-suspended in 400 μl 1 × binging buffer, and analyzed by using FACScan flowcytometer (Becton Dickinson, San Jose, CA, USA).

***Cell transfection***

In order to suppress miR-454 expression, the vector of miR-454 inhibitor was synthesized by GenePharma Co. (Shanghai, China), and transfected into H9c2 cells. The negative control (NC) served as a blank control group. All cell transfections were conducted by using Lipofectamine 3000 reagent (Invitrogen) based on the manufacturer’s protocol. After transfection for 48 hr, the cells were harvested for the following researches.

***Quantitative real-time polymerase chain reaction (qRT-PCR)***

After treatment with OGD or Pioglitazone, the Trizol reagent (Life Technologies Corporation, Carlsbad, CA, USA) was used to extract the total RNA of treated H9c2 cells according to the kit instructions. Taqman MicroRNA Reverse Transcription Kit (TaKaRa, Dalian, China) was used to reverse transcribe the RNA sample into cDNA. The Taqman Universal Master Mix II with the TaqMan MicroRNA Assay (Applied Biosystems, Foster City, CA, USA) was used to detect miR-454 expression in these treated cells. U6 was used as a loading control. The data was measured by using the 2^-ΔΔCT^ method (22). The specific primer sequence for miR-454 was as Forward: 5’-GGGACCCTATCAATATTGT-3’ and Reverse: 5’- CAGTGCGTGTCGTGGAGT-3’. U6 primer sequence was as Forward: 5’-CTTCGGCAGCACATATACT-3’ and Reverse: 5’-AAAATATGGAACGCTTCACG-3’. 

***Western blot***

The proteins of H9c2 cells with different treatments were extracted using RIPA lysis buffer (Beyotime Biotechnology, Shanghai, China) supplemented with protease inhibitors (Roche, Basle, Switzerland). The BCA Protein Assay Kit was used to analyze and determine the total protein concentration. Protein samples (50 μg) were electrophoresed by SDS-PAGE. Then, these proteins were transferred to PVDF membranes (Millipore, Billerica, MA, USA), and these membranes were placed in 5% (w/v) non-fat milk and shaken for 1 hr at room temperature. The blocked membranes were shake-washed in Tris-buffered saline with 0.1 % (v/v) Tween 20 (TBST) for 10 min. After this, the membranes were incubated with the following diluted primary antibodies at 4°C overnight: Cyclin D1 (ab16663, 1:200 dilution), p21 (ab109199; 1:1000 dilution), Bax (ab53154, 1:500 dilution), pro-Caspase-3 (ab205733, 1:5000 dilution), cleaved-Caspase-3 (ab32042; 1:500 dilution), pro-Caspase-9 (ab138412; 1:1000 dilution), cleaved-Caspase-9 (ab2324; 1:1000 dilution), t-PI3K (ab191606, 1:1000 dilution), p-PI3K (ab182651, 1:500 dilution), t-AKT (ab8805, 1:500 dilution), p-AKT (ab8933, 1:500 dilution) and β-actin (ab8227, 1:1000 dilution) (All from Abcam, Cambridge, UK). 

**Figure 1:**
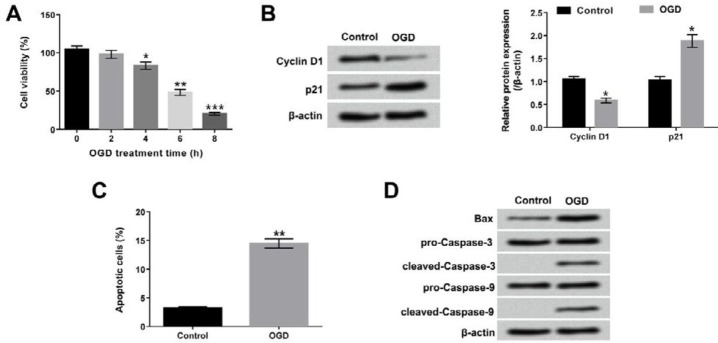
Oxygen glucose deprivation (OGD) treatment induced H9c2 cells injury. (A) H9c2 cells were maintained under OGD for 0, 2, 4, 6 and 8 hr, and the viability of H9c2 cells was subsequently analyzed by cell counting kit-8 (CCK-8) assay. After treatment of OGD for 6 hr, (B) Western blot assay was performed to detect the protein levels of Cyclin D1 and p21 in OGD-treated cells; (C) flow cytometry with Annexin V-FITC/PI staining was performed to measure the percentage of apoptotic cells; (D) the apoptosis-associated protein levels were assessed by Western blot. *P< 0.05; **P< 0.01; ***P< 0.001

**Figure 2:**
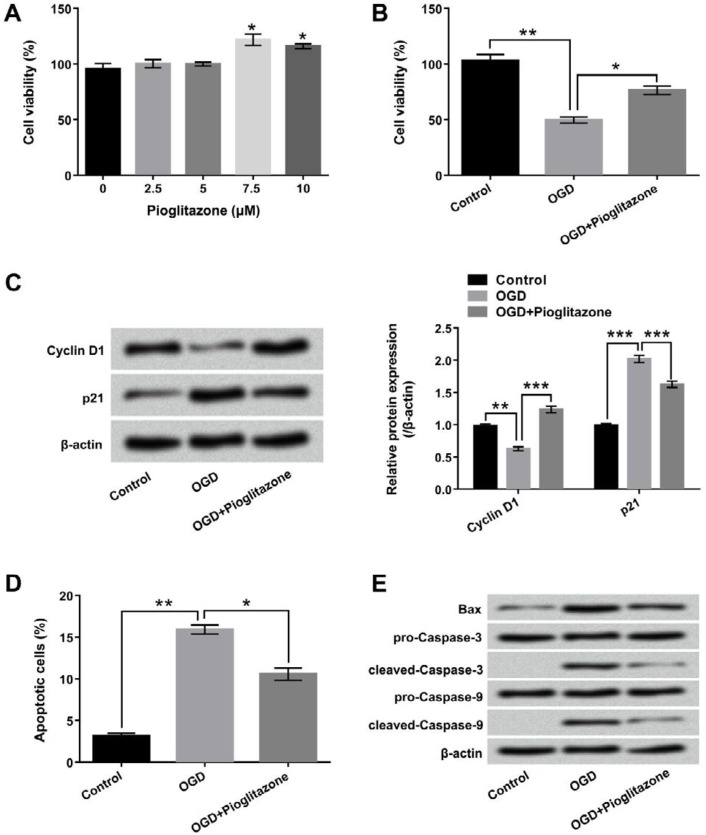
Pioglitazone attenuated oxygen glucose deprivation (OGD)-induced H9c2 cells injury. (A) H9c2 cells were stimulated with indicated concentrations of pioglitazone (0, 2.5, 5. 7.5 and 10 μM) for 12 hr, and then cell viability was examined by cell counting kit-8 (CCK-8). H9c2 cells were pretreated with 5 μM of pioglitazone for 12 hr, and were maintained under OGD for 6 hr, (B) cell viability (C) Cyclin D1 and p21 protein levels were determined by CCK-8 and Western blot assays; (D) cell apoptosis and (E) apoptosis-related protein levels were analyzed by flow cytometry with Annexin V-FITC/PI staining and Western blot. *P< 0.05; **P< 0.01; ***P< 0.001

**Figure 3:**
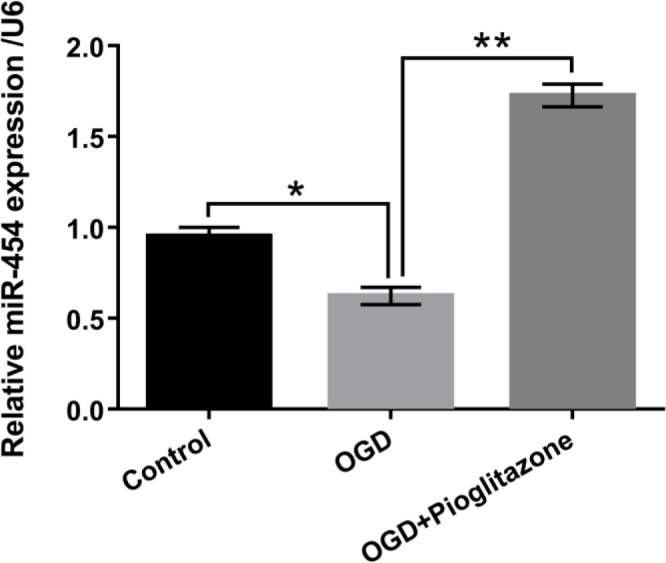
Pioglitazone up-regulated miR-454 expression in oxygen glucose deprivation (OGD)-injured H9c2 cells. H9c2 cells were pretreated with 5 μM of pioglitazone for 12 hr and were maintained under OGD for 6 hr; miR-454 expression level was measured by qRT-PCR. *P< 0.05; **P< 0.01

After incubation and washing, the membranes were incubated with goat anti-rabbit secondary antibody conjugated with horseradish peroxidase (ab205781, 1:5000 dilution, Abcam) for 1 hr at room temperature. Subsequently, the membranes were tiled on the preservative film, and the ECL Western blotting reagent mixture (Pierce Biotechnology, Thermo Fisher Scientific Inc., Waltham, MA, USA) was uniformly dropped on the membranes. After reaction, the experiment figures were analyzed by using Image Lab™ Software (Bio-Rad, Shanghai, China). 

**Figure 4:**
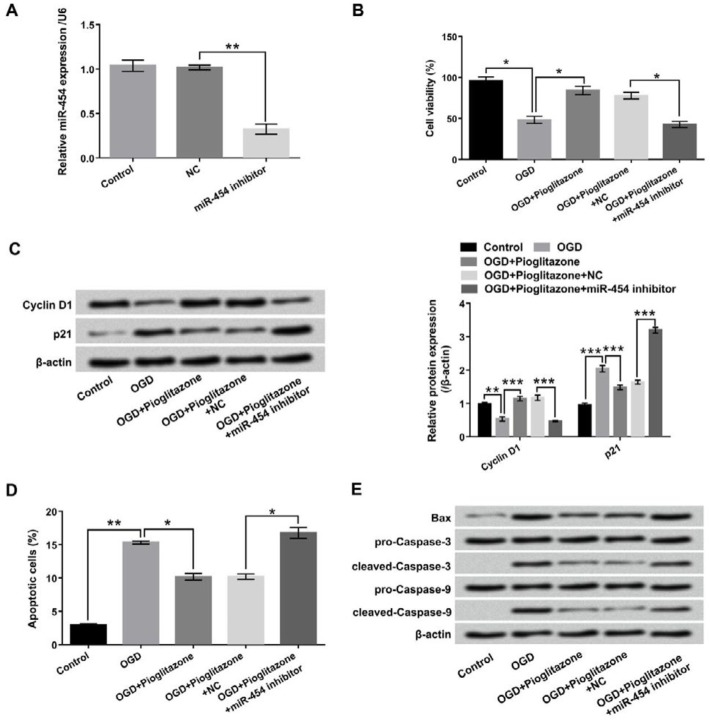
Pioglitazone alleviated oxygen glucose deprivation (OGD)-induced H9c2 cells injury through up-regulating miR-454. The expression vectors of miR-454 inhibitor and its negative control (NC) were transfected into H9c2 cells, and then (A) the expression level of miR-454 was detected by qRT-PCR after treatment with 5 μM of pioglitazone for 12 hr and were maintained under OGD for 6 hr, (B) cell viability (C) Cyclin D1 and p21 protein levels were determined by cell counting kit-8 (CCK-8) and Western blot; flow cytometry with Annexin V-FITC/PI staining and Western blot assay were performed to analyze (D) cell apoptosis and (E) the protein levels of apoptosis-related factors. *P< 0.05; **P< 0.01; ***P< 0.001

**Figure 5:**
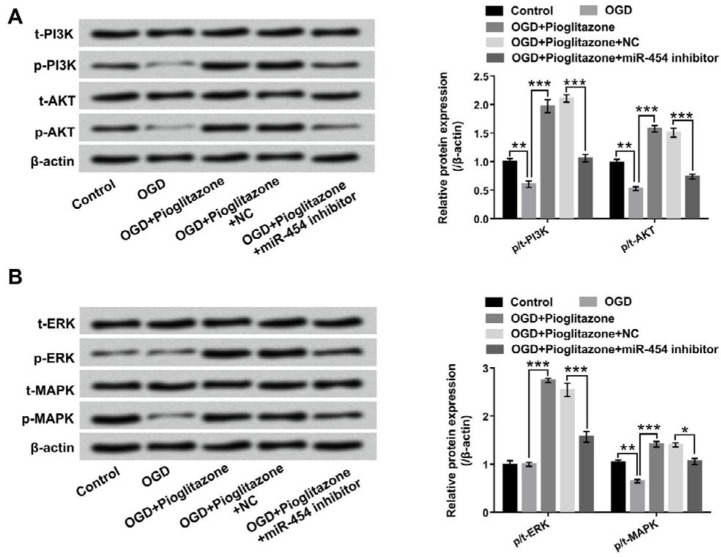
Pioglitazone activated phosphoinositide 3-kinase (PI3K/)/protein kinase B (AKT) and extracellular signal-regulated kinases (ERK)/mitogen-activated protein kinase (MAPK) signal pathways via regulation of miR-454. H9c2 cells were transfected with miR-454 inhibitor and negative control (NC), and the transfected cells were pretreated with 5 μM of pioglitazone for 12 hr and were maintained under oxygen glucose deprivation (OGD) for 6 hr; the protein levels of (A) p/t-PI3K and p/t-AKT as well as (B) p/t-ERK and p/t-MAPK were determined by Western blot. *P< 0.05; **P< 0.01; ***P< 0.001

The gray value of the blots in the figures reflected the expression level of the target proteins.

***Statistical analysis***

All data from this study are shown as the mean ± standard deviation (SD). SPSS statistical software version 19.0 (IBM, Armonk, NY, USA) was used for statistical analyses. One-way analysis of variance (ANOVA) was used to calculate the *P-* values. The difference between groups with *P*<0.05 was considered as a statistically significant result.

## Results

***Construction of OGD-induced H9c2 cells injury model in vitro***

A cell model of OGD-induced injury in H9c2 cells was constructed. Results showed that the viability of H9c2 cells was significantly decreased when cells had undergone OGD at 4 hr, 6 hr and 8 hr (*P*<0.05, *P*<0.01 or *P* < 0.001, Figure 1A). Moreover, Cyclin D1 protein level was down-regulated and p21 protein level was up-regulated after OGD treatment (*P*<0.05, Figure 1B). The percentage of apoptotic cell was prominently induced in OGD-treated cells compared to non-treated cells (*P* < 0.01, Figure 1C). Meanwhile, the protein levels of apoptosis-associated factors of Bax and cleaved-Caspase-3/-9 were promoted under OGD treatment (Figure 1D). Furthermore, the treatment time of OGD for 6 hr was selected for the following experiments.

***Pioglitazone attenuated OGD-induced H9c2 cells injury***

H9c2 cells were stimulated with the different concentrations of pioglitazone (0-10 μM), and cell viability was examined. As shown in Figure 2A, pioglitazone significantly promoted cell viability at the concentrations of 7.5 μM and 10 μM (*P*<0.05). But, pioglitazone had no effect on cell viability at the concentrations of 2.5 μM and 5 μM. Therefore, 5 μM pioglitazone was selected as the best treatment concentration in subsequent expressions. In Figure 2B, the results displayed that cell viability was significantly promoted by pioglitazone after OGD treatment (*P*<0.05). 

Figure 2C revealed that pioglitazone obviously up-regulated the protein level of Cyclin D1 and down-regulated the protein level of p21 after OGD treatment (*P* < 0.001). Additionally, the results in Figure 2D and 2E showed that pioglitazone significantly suppressed OGD-induced cell apoptosis (*P* < 0.05). The protein levels of Bax and cleaved-Caspase-3/-9 were decreased by Pioglitazone after OGD treatment. 

***Pioglitazone up-regulated miR-454 expression in OGD-treated H9c2 cells***

As result displayed in Figure 3, miR-454 expression in OGD-treated cells was significantly lower than that in non-treated cells (*P*<0.05). However, miR-454 expression level was significantly increased after adding the pioglitazone on OGD-treated H9c2 cells (*P*<0.01). Above data suggested the probable involvement of miR-454 in the protective effect of pioglitazone against OGD-induced injury in H9c2 cells.

***Pioglitazone attenuated OGD-induced H9c2 cells injury via up-regulation of miR-454***

To further explore whether miR-454 was participated in mediating OGD-induced H9c2 cells injury, miR-454 inhibitor was transfected into H9c2 cells to regulate miR-454 expression. In Figure 4A, qRT-PCR analytical result showed that the expression of miR-454 was significantly decreased by miR-454 inhibitor, indicating well transfection efficiency (*P*<0.01). Next, the results in Figure 4B revealed that miR-454 suppression significantly decreased cell viability after co-treatment of OGD and pioglitazone (*P*<0.05). The protein level of Cyclin D1 was down-regulated by miR-454 suppression; as well, the protein level of p21 was up-regulated by miR-454 suppression after treatment of Pioglitazone in OGD-injured H9c2 cells (*P*<0.001, Figure 4C). Furthermore, miR-454 suppression remarkably promoted cell apoptosis, meanwhile up-regulated Bax and cleaved-Caspase-3/-9 expressions after treatment of pioglitazone in OGD-injured H9c2 cells (*P*<0.05, Figure 4D and 4E). 

***Pioglitazone activated PI3K/AKT and ERK/MAPK signal pathways by regulation of miR-454***

Western blot assay was performed to determine the functions of pioglitazone in PI3K/AKT and ERK/MAPK signal pathways. The results in Figure 5A and 5B showed that the protein levels of p-PI3K, p-AKT, p-ERK and p-MAPK were remarkably increased by pioglitazone after OGD treatment (*P*< 0.001). However, the promoting effects of pioglitazone on these two signal pathways were obviously declined by miR-454 suppression (*P*< 0.05 or *P*<0.001). The protein levels of t-PI3K, t-AKT, t-ERK and t-MAPK had no significant changes in the different treatment groups. 

## Discussion

In our study, the model of OGD**-**induced H9c2 cell injury was successfully constructed. Then, we observed that Pioglitazone significantly promoted cell proliferation and reduced apoptosis in OGD-injured H9c2 cell. However, the protective effects of Pioglitazone on OGD-injured H9c2 cell were abolished by suppression of miR-454. Furthermore, the results revealed that Pioglitazone activated PI3K/AKT and ERK/MAPK signal pathways by up-regulation of miR-454 in OGD-injured H9c2 cells.

OGD-induced cell injury is a complicated process that accompanies the generation of reactive oxygen species (ROS), calcium (Ca^+^) overloading and mitochondrial permeability transition pore (mPTP) opening (23). Recent study proved that OGD has been widely used to treat cardiomyocytes and to construct cell model of ischemic heart damage (24). The model of OGD-induced MIRI could be used to explore the molecular mechanisms of MIRI and screen effective drugs to alleviate MIRI (25). Additionally, mounting evidences displayed that H9c2 cardiomyocytes were broadly used for investigating the protective effects of agents on OGD-induced ischemic heart injury (23, 26). Based on these studies, we constructed an OGD-induced H9c2 cells injury model to investigate the effect of Pioglitazone on MIRI. We found that cell viability and cell cycle-related protein levels were decreased, while cell apoptosis and the protein levels of apoptosis-related factors were increased in OGD-treated H9c2 cells. These data stated that the OGD-induced H9c2 cells injury model was successfully constructed *in vitro*.

Pioglitazone is an anti-diabetic agent in the thiazolidinedione class, which has been used to treat several diseases, including MIRI (27). Recent studies have demonstrated that Pioglitazone could protect heart and alleviate MIRI by suppressing cell apoptosis (28). An animal experiment revealed that pharmacological preconditioning with nicorandil and Pioglitazone could alleviate MIRI in rats (29). However, whether Pioglitazone could exert the protective effect on OGD-injured H9c2 cells has not been clarified. In the present study, we found that pioglitazone alleviated OGD-induced H9c2 cells injury by restoring cell viability and proliferation-associated factors expressions and declined apoptosis in OGD-treated H9c2 cells, indicating the protective role of pioglitazone in OGD-injured H9c2 cells.

Recent evidences have proven that various miRNAs such as miR-214, miR-22 and miR-103/107 are involved in regulation of MIRI (30-32). MiR-454 functions as an oncogene has been confirmed in various cancers, such as hepatocellular and colorectal cancer (19, 33). Study from Tao *et al*. revealed that miR-454 alleviated lipopolysaccharides (LPS)-induced acute lung injury (ALI) in lung epithelial cells (20). However, the effect of miR-454 on MIRI has not been investigated. In our study, miR-454 inhibitor was transfected into H9c2 cells to alter miR-454 expression. Results in this study revealed that miR-454 expression was up-regulated by Pioglitazone. However, miR-454 suppression abolished the protective effect of pioglitazone on OGD-injured H9c2 cells. These data suggested that pioglitazone alleviated OGD-induced H9c2 cells injury through up-regulation of miR-454.

PI3K/AKT and ERK/MAPK signal pathways are vital regulators in cell growth and inflammatory response (34, 35). Several studies have proven that activation of PI3K/AKT and ERK/MAPK signal pathways plays critical role in the protection against MIRI (36, 37). Taniguchi *et al*. uncovered that pioglitazone could alleviate MIRI through increasing the expression of heat shock protein 72 (HSP72), and activating PI3K/AKT signal pathway (38). Another study found that pioglitazone could protect cardiomyocytes against I/R-induced apoptosis by regulation of PI3K/ERK1/2 signal pathway (39). Similar with these studies, we found that pioglitazone could activate PI3K/AKT and ERK/MAPK signal pathways by regulation of miR-454. These findings indicated that activation of these two signal pathways might contribute to the reduction of MIRI.

## Conclusion

Taken together, the results from present study suggested that pioglitazone could protect H9c2 cells against OGD-induced injury by activation of PI3K/AKT and ERK/MAPK signal pathways through up-regulating miR-454. These findings might provide a preliminary pharmacological basis for pioglitazone in the treatment of MIRI. 

## Conflict of Interest

The authors declare that they have no competing interests.
